# SARS-CoV-2 is associated with high viral loads in asymptomatic and recently symptomatic healthcare workers

**DOI:** 10.1371/journal.pone.0248347

**Published:** 2021-03-18

**Authors:** M. Catherine McEllistrem, Cornelius J. Clancy, Deanna J. Buehrle, Nina Singh, Aaron Lucas, Valerie Sirianni, Brooke K. Decker

**Affiliations:** 1 Department of Medicine, Infectious Diseases Section, VA Pittsburgh Healthcare System, Pittsburgh, PA, United States of America; 2 Department of Medicine, Division of Infectious Diseases, University of Pittsburgh, Pittsburgh, PA, United States of America; Columbia University, UNITED STATES

## Abstract

**Background:**

Healthcare workers (HCW) are at increased risk of SARS-CoV-2 infection from both patients and other HCW with coronavirus disease 2019 (COVID-19). RT-PCR cycle threshold (Ct) values of SARS-CoV-2 ≤ 34 and the first 7–9 days of symptoms are associated with enhanced infectivity. We determined Ct values and duration of symptoms of HCW with a positive SARS-CoV-2 test. As HCW often assume their greatest risk of acquiring SARS-CoV-2 is working on a COVID-19 unit, we also determined Ct values and symptom duration of inpatients with a positive SARS-CoV-2 test.

**Methods:**

From 6/24/2020-8/23/2020, Ct values and duration of symptoms from 13 HCW, 12 outpatients, and 28 inpatients who had a positive nasopharyngeal swab for SARS-CoV-2 were analyzed.

**Results:**

Among HCW with a positive SARS-CoV-2 test, 46.2% (6/13) were asymptomatic and requested testing due to an exposure to someone with COVID-19; 83.3% (5/6) of those exposures occurred in the community rather than in the hospital. The median Ct value of HCW was 23.2, and 84.6% (11/13) had a Ct value ≤ 34. The median Ct value of 29.0 among outpatients with COVID-19 did not significantly differ from HCW. In contrast, inpatients with a positive SARS-CoV-2 test had a median Ct value of 34.0 (p = 0.003), which translated into a median ~1,000-fold lower viral load than observed in HCW. Among those with symptoms related to COVID-19, no (0/6) HCW compared to 50% (6/12) of inpatients had symptoms for at least one week (p = 0.04).

**Conclusions:**

At our institution, asymptomatic COVID-19 accounted for nearly half of the cases among HCW. Symptomatic HCW had high viral loads and short duration of symptoms, both of which are associated with peak infectivity. Infection prevention programs should educate HCW on these findings in an effort to increase adherence to the requirement to maintain six feet separation in workspaces and breakrooms, in addition to consistently wearing personal protection equipment.

## Introduction

Health care workers (HCW) can contract severe acute respiratory syndrome coronavirus 2 (SARS-CoV-2), the agent responsible for coronavirus disease 2019 (COVID-19) from hospitalized patients, with increased risk associated with unprotected, prolonged patient contact and aerosol-generating procedures [1]. Among ~3000 HCW at 13 academic centers, 6% had antibody evidence of previous SARS-CoV-2 infection. Masks are protective, as the prevalence of SARS-CoV-2 antibodies was 5.6% among HCW who always wore masks compared to 9.0% of HCW who did not [2]. Within the hospital, the SARS-CoV-2 positivity rate was 8.3% among HCW who worked in COVID-19 units and 3.4% among those who worked elsewhere in the hospital [3]. In another study, 3% of ~1,000 asymptomatic HCW had a positive SARS-CoV-2 test [4]. Transmission among HCW has been linked to the presence of a SARS-CoV-2 positive person in their household, use of the hospital break rooms without a medical mask for more than 15 minutes, consuming food within one meter of another HCW, and failure to keep a safe social distance from an HCW [3]. Among those with COVID-19, infectivity has been associated with the first 7–9 days of symptoms [5–7], and appears most likely between days 1–5 [5].

The real-time reverse transcription-PCR (RT- PCR) cycle threshold value (Ct value) has an inverse correlation to the amount of SARS-CoV-2 present. For every one unit increase in Ct, the odds ratio of infectivity decreases by 32% [5]. When the Ct value rises above 34, live virus has not been detected *in vitro* [6, 7]. In this study, we characterized the Ct values and duration of symptoms among HCW with a positive SARS-CoV-2 test at the Veterans Affairs Pittsburgh Healthcare System (VAPHS). This hospital is located in Allegheny County, a region of Pennsylvania that had a low prevalence of COVID-19 during this time period [8]. As HCW often assume their greatest risk of acquiring SARS-CoV-2 is working on a COVID-19 unit, we compared Ct values and symptom duration with inpatients with a positive SARS-CoV-2 test. At our institution, all inpatients undergo pre-admission SARS-CoV-2 testing.

## Materials and methods

### Patient and healthcare worker characteristics

From 6/24/2020-8/23/2020, we collected clinical and laboratory data on both patients and HCW with a positive RT-PCR test for SARS-CoV-2 from a nasopharyngeal sample using the Cepheid Xpert Xpress SARS-CoV-2 test system. Outpatients were classified as patients with a positive SARS-COV-2 PCR obtained in the emergency department or an outpatient clinic who were not admitted to the hospital. Inpatients were classified as patients with a positive SARS-COV-2 PCR obtained in the emergency department who were admitted to an inpatient ward. Healthcare workers were classified as employees with a positive SARS-COV-2 PCR test. Inpatients and outpatients were tested for SARS-CoV-2 by the emergency room staff; employees were tested by either the occupational health staff or emergency room staff.

Co-morbid conditions were collected on all inpatients, and consisted of diseases associated with severe COVID-19: active cancer, chronic kidney disease, chronic obstructive pulmonary disease, solid organ transplant, obesity (body mass index ≥ 30), congestive failure/coronary artery disease/cardiomyopathies, diabetes mellitus, and sickle cell disease. Symptomatic infection was defined as the presence of new or worsening symptoms associated with COVID-19. Patients and HCW at VAPHS were required to follow the Centers for Disease Control and Prevention (CDC) guidelines regarding use of personal protection equipment (PPE) in the healthcare setting; moreover, all HCW must answer screening questions regarding COVID-19 symptoms and exposure history in addition to having their temperature checked before entering the hospital. Inpatients are encouraged but not required to wear a mask when within six feet of another person. This research was completed as a quality improvement project, and no participants were recruited. We included patients with an initial positive SARS-COV-2 PCR result from the Cepheid Xpert Xpress system. Given that Ct values can vary between platforms, patients who had a nasopharyngeal swab processed with a different RT-PCR system were excluded. Only the first nasopharyngeal specimen was used in the case of duplicates. Hospital systems that screen everyone for COVID-19 upon entry to their building, and perform a nasopharyngeal SARS-CoV-2 test prior to inpatient admission would be expected to have similar results.

### Laboratory data/statistical analyses

The RT-PCR Ct value was determined for the Envelope (E) and nucleocapsid (N2) gene targets of SARS-CoV-2. A negative result was defined as the absence of both PCR amplification products or single-primer positive for E target after 45 cycles. The Cepheid system has a viral limit of detection of < 100 viral copies/ ml [9]. The viral load can be extrapolated from the Ct value, with each Ct value being 2-fold different through log base 2 calculations. Fisher’s exact test was used to evaluate the categorical variables, and the two-tailed t-test was used to compare continuous variables.

## Results

From 6/24/20-8/23/20, 53 patients and HCW at the VAPHS had a nasopharyngeal swab positive for SARS-CoV-2 by RT-PCR: 13 HCW, 28 inpatients, and 12 outpatients. Reported symptoms of COVID-19 included fever, cough, shortness of breath, myalgia, headache, fatigue, anosmia, dysgeusia, and diarrhea. For 20.8% (11/53) of the individuals, the E gene target was not detected after 45 cycles of RT-PCR; thus, the Ct value for the N2 gene target was used in the analyses. The Ct values for the N2 genetic marker were determined for all 53 individuals and ranged from 16.9 to 44.5.

### Healthcare workers

Among HCW, 46.2% (6/13) were asymptomatic and requested testing due to an exposure to someone with COVID-19; 83.3% (5/6) of those exposures occurred in the community rather than in the hospital (Table 1). HCW included patient care providers, cleaning personnel, food service employees, phlebotomists, and technicians from laboratory, radiology, and pharmacy services.

**Table 1:** Demographic characteristics and RT-PCR Cycle threshold (Ct) values of healthcare workers (HCW), inpatients, and outpatients.

	Outpatients (N = 12)	Healthcare Workers (HCW) (N = 13)	Inpatients (N = 28)	HCW vs. Inpatients	Inpatients vs. Oupatients	HCW vs. Outpatients
				P value	P value	P value
Median age (IQR) ^a^	51.5 (42.3–68.3)	51 (40.0–58.0)	72.5 (61.5–77.8)	0.00005	0.004	0.32
At least one co-morbid condition–n/N (%)	9 (75.0)	N/A^b^	24 (85.7)	N/A^b^	0.41	N/A^b^
Median Ct value ^c^ (IQR) ^a^	29.0 (25.2–35.2)	23.2 (19.9–25.3)	34.0 (27.9–41.4)	0.003	0.09	0.26
Ct value ^c^ ≤34 –n/N (%)	8 (66.7)	11 (84.6)	15 (53.6)	0.08	0.50	0.38

^a^ IQR, interquartile range.

^b^ N/A not available.

^c^ Cycle threshold value for N2 primer target of SARS-CoV-2.

Eighty-five percent (11/13) of HCW had a Ct value ≤34 (Table 1). The median Ct value of HCW was 23.2 (interquartile range, 19.9–25.3; Table 1), with median Ct value of 25.3 (20.8–27.0, interquartile range,) among asymptomatic, and 23.2 (21.1–23.4) among symptomatic individuals (Table 2). Among symptomatic HCW, the median duration of symptoms was 3 days (interquartile range, 1–3; Table 2), and none (0/6) of HCW had symptoms for ≥ 7 days (Table 2).

**Table 2:** Comparison of asymptomatic and symptomatic persons with a positive SARS-CoV-2 test.

		Outpatients	Healthcare Workers (HCW)	Inpatients	HCW vs. Inpatients	Inpatients vs. Oupatients	HCW vs. Outpatients
					P value	P value	P value
Asymptomatic		2/12 (16.7)	6/13 (46.2)	13/28 (46.2)	1.0	0.15	0.20
n/N (%)							
Ct ^a^ Median (IQR)	Asymptomatic [21 individuals]	ND ^b^	25.3 (20.8–27.0)	41.3 (34.0–41.7)	0.001	ND ^b^	ND ^b^
	Symptomatic [32 individuals]	27.7 (22.2–34.1)	23.2 (21.1–23.4)	28.2 (25.9–34.4)	0.18	0.59	0.45
Ct ^a^ ≤34	Asymptomatic [21 individuals]	ND ^b^	5/6 (83.3)	4/13 (30.8)	0.02	ND ^b^	ND ^b^
n/N (%)							
	Symptomatic [32 individuals]	7/10 (70.0)	6/7 (85.7)	11/15 (73.3)	0.64	1.0	0.60
Median Symptom (IQR)		3 (1–3)	3 (1–3)	9 (4–14)	0.04	0.37	0.33
Duration [29 individuals] ^c^							
Duration ≥ 7 days		1/10 (10)	0/7 (0)	6/12 (50)	0.04	0.07	1.0
(29 individuals) ^c^							
n/N (%)							
Duration ≥ 10 days		1/10 (10)	0/7 (0)	5/12 (42)	0.11	0.16	1.0
(29 individuals) ^c^							
n/N (%)							

^a^ Cycle threshold.

^b^ Only 2 individuals.

^c^ 15 inpatients with symptoms, but 3 with unknown duration.

### Patients

The median age of inpatients was 72.5 years, which was significantly higher than both outpatients (51.5 years) and HCW (51.0 years) (p = 0.00005; Table 1). Among inpatients, 82.1% (23/28) were ≥ 55 years old and 67.9% (19/28) were ≥ 65 years old. The proportion of inpatients and outpatients who had at least one co-morbid condition associated with severe COVID-19 disease did not significantly differ: 85.7% (24/28) of inpatients and 75% (9/12) of outpatients. Among inpatients, 46.2% (13/28) were asymptomatic and were admitted for unrelated reasons; the test was only completed as a requirement for admission. An additional 10.7% (3/28) of inpatients had severe dementia and were admitted with shortness of breath and hypoxia of unknown duration.

**Fig 1:**
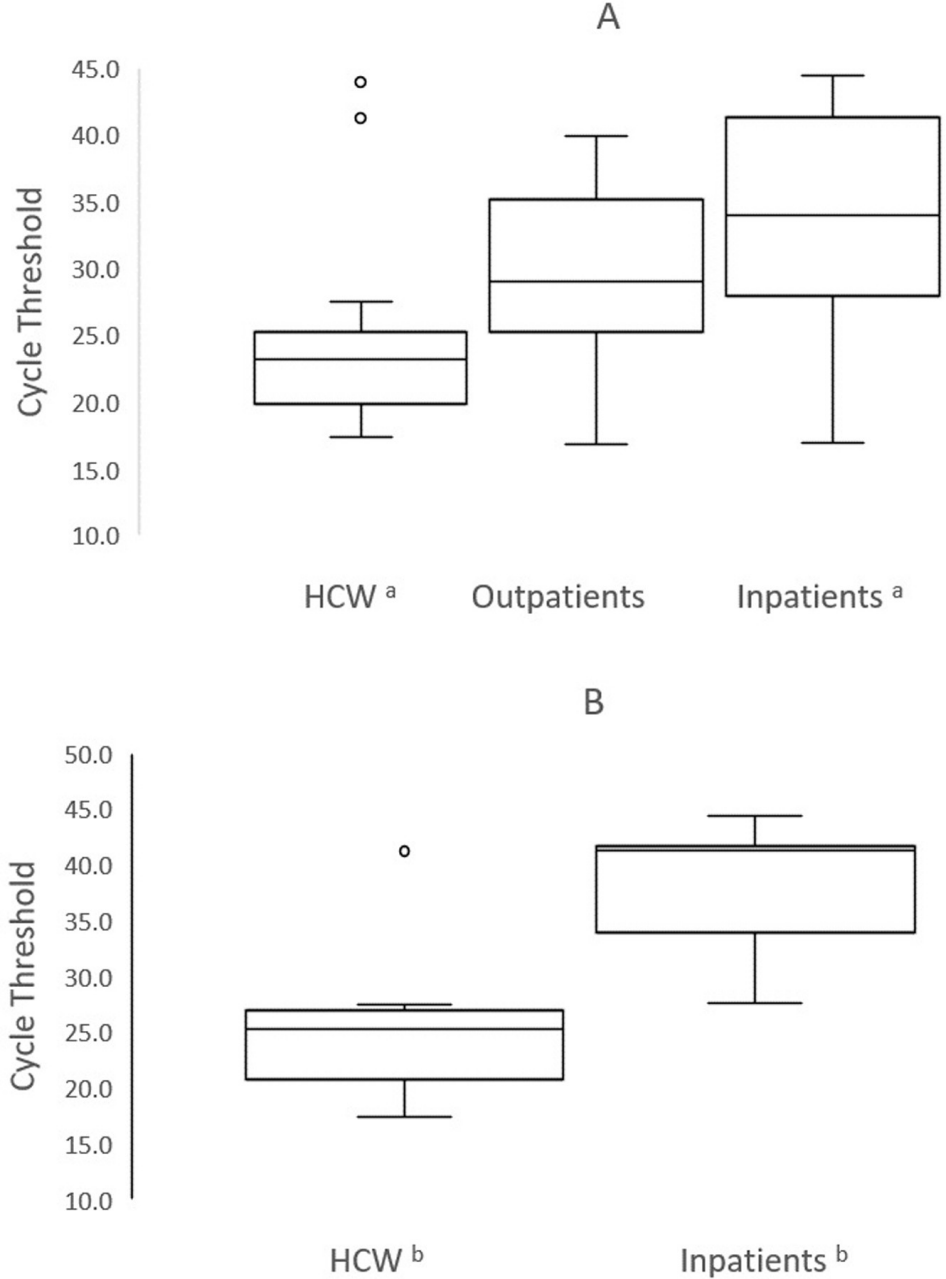
Comparison of the cycle threshold values of healthcare workers (HCW), outpatients, and inpatients with a positive RT-PCR SARS-CoV-2 test. A, All individuals ^a^ p = 0.003. B, Asymptomatic individuals ^b^ p = 0.001. Midlines indicate the median, boxes indicate interquartile ranges, whiskers indicate the upper and lower adjacent values (within 1.5-fold the interquartile range), and isolated data points indicate outliers.

**Fig 2:**
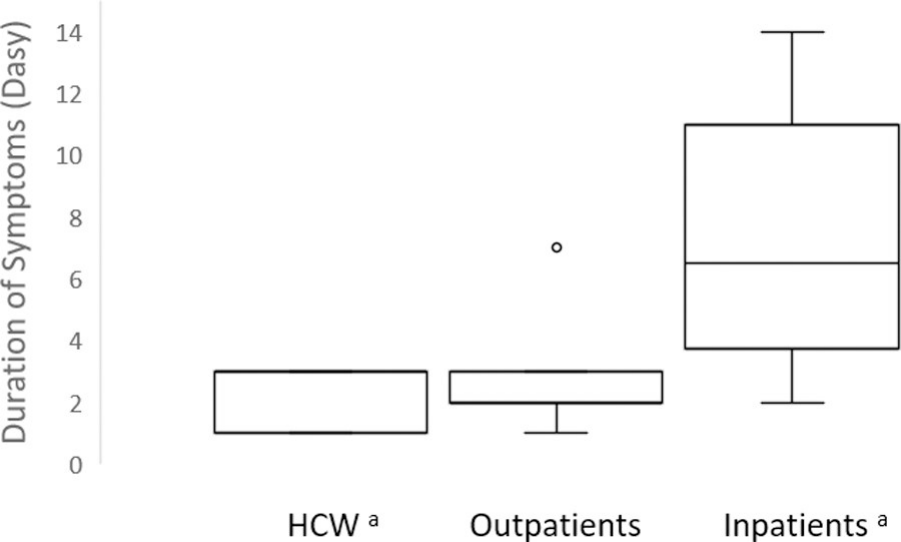
Duration of symptoms prior to symptom duration prior to a positive SARS-CoV-2 test among Heathcare Workers (HCW), outpatients, and inpatients, ^a^ p value 0.04. A, One outpatient had symptoms for 30 days; One inpatient had symptoms for 28 days. Midlines indicate the median, boxes indicate interquartile ranges, whiskers indicate the upper and lower adjacent values (within 1.5-fold the interquartile range), and isolated data points indicate outliers.

The median Ct value of HCW was significantly lower compared to inpatients: 23.2 (interquartile range, 19.9–25.3) for HCW and 34.0 (interquartile range, 27.9–41.4) for inpatients (p = 0.003; Fig 1A and Table 1). This difference in Ct value translated into a median ~1,000-fold higher viral load in HCW compared to inpatients. In contrast, the Ct values among outpatients, with a median of 29.0 (interquartile range, 25.2–35.2), and HCW did not significantly differ (Fig 1A and Table 1). Among asymptomatic individuals, HCW had a median Ct value of 25.3 (interquartile range, 20.8–27.0) while inpatients had a median Ct value of 41.3 (interquartile range, 34.0–41.7; p = 0.001; Table 2 and Fig 1B;). Likewise, among asymptomatic persons, 83.3% (5/6) of HCW compared to 30.8% (4/13) of the inpatients had a Ct value ≤34 (p = 0.02; Table 2). The median duration of symptoms was significantly longer in inpatients compared to HCW: 9 days among inpatients versus 3 days among HCW (p = 0.04; Table 2 and Fig 2). None (0/6) of HCW compared to 50% (6/12) of the inpatients had symptoms for ≥ 7 days (p = 0.04; Table 2).

## Discussion

While other studies have demonstrated the importance of asymptomatic infections among HCW [4], to our knowledge this is the first study to fully characterize Ct values and duration of symptoms among HCW with a positive SARS-CoV-2 test. Eighty-five percent (11/13) had a Ct value ≤34 at the time of diagnosis, a value associated with infectivity [6, 7]. Nearly half of the HCW were asymptomatic, with the majority requesting a SARS-CoV-2 test due to a COVID-19 exposure in the community. Among HCW with COVID-19 symptoms, all had symptoms for less than a week, a time period associated with peak transmission [5–7].

HCW use extreme caution when caring with patients on the COVID-19 units; however, consistent use of PPE and adherence to distancing guidelines when interacting with their peers in their workspaces and breakrooms is needed to prevent transmission of COVID-19 between HCW as well. While comparisons between HCW and inpatients are associated with inherent limitations, these analyses were performed in an effort to illustrate the important role that peer-to-peer HCW interactions could play in COVID-19 transmission. We found that HCW with a positive SARS-CoV-2 test had significantly lower median Ct values than inpatients treated on the COVID-19 units. Given the 2-fold change in viral load associated with a one-point difference in Ct value, this resulted in a ~1,000-fold higher viral load in HCW compared to inpatients. Moreover, among those who were asymptomatic from COVID-19, HCW were more likely than inpatients to have Ct values ≤ 34, a level associated with infectivity [6, 7]. In addition, HCW had COVID-19 symptoms for a shorter duration prior to testing compared to inpatients.

The rationale for testing for SARS-CoV-2 likely explains the variation between in Ct values HCW and inpatients. The asymptomatic HCW were tested due to a recent exposure to someone with COVID-19, and the symptomatic HCW were tested for even mild COVID-19 symptoms or documented elevated temperature. In contrast, the asymptomatic inpatients were tested as a pre-requisite to admission, and the symptomatic inpatients only presented to the emergency room when their COVID-19 symptoms were ongoing and unmanageable at home. As the viral load decreases over the course of infection [10], the timing of the test may explain these findings. However, this interpretation may be too simplistic, as older age, the presence of co-morbidities and severe disease have been linked to low Ct values [6, 11, 12]. Moreover, other factors may have accounted for the difference detected in Ct value and symptom duration prior to a positive SARS-CoV-2 test in this study. First, the NP collection technique could conceivably impact the Ct value as separate hospital departments obtained the samples for HCW and patients. Second, it is unknown whether intensity and duration of exposure of the virus could account for the variation. Further studies on the association between age and co-morbidities with Ct value and symptom duration are needed. As well, this study did not assess transmission to HCW performing aerosol-generating procedures in critically ill patients on the COVID-19 units. Further research is needed to establish the threshold viral load necessary for transmission in this setting.

Despite these limitations, these data support the need for infection prevention programs to focus on workspace and breakrooms [3]. In South Korea, prolonged exposure to an employee infected with SARS-CoV-2 resulted in an employee attack rate of 44% [13]. Based on CDC guidelines, policies to avoid HCW congregation and ensure workstation social distancing of at least 6 feet is prudent. To increase adherence, healthcare facilities should consider removing extra chairs and clearly marking appropriately distanced workstation locations. The workspaces, breakrooms, and cafeterias used by HCW should have frequent cleaning of high touch and communal equipment. To accommodate space restrictions, breaks should be staggered, and meeting rooms repurposed. As well, outside dining options should be considered when possible. Given the possible ineffectiveness of gaiters, loose bandanas, and valved face masks [14], standards for HCW face coverings should be developed using only those with demonstrated efficacy.

Since HCW cases in this study were usually associated with community COVID-19 exposure, rising rates should trigger surveillance testing of HCW as resources allow. As viral load has been linked to duration of symptoms [5, 10], the detection of only high Ct values among HCW tested for SARS-CoV-2 could suggest that delayed testing after exposure or symptom onset may be occurring. In this case, education efforts could be redoubled to encourage HCW to undergo early SARS-CoV-2 testing when appropriate. Based on our current understanding, these workplace interventions could lead to a reduced risk of transmission of SARS-CoV-2 between HCW.
